# Mechanisms of Acute Kidney Injury–Chronic Kidney Disease Transition: Unraveling Maladaptive Repair and Therapeutic Opportunities

**DOI:** 10.3390/biom15060794

**Published:** 2025-05-29

**Authors:** Dongxue Xu, Xiaoyu Zhang, Jingjing Pang, Yiming Li, Zhiyong Peng

**Affiliations:** 1 Department of Critical Care Medicine, Zhongnan Hospital of Wuhan University, Wuhan 430071, China; xudongxue@whu.edu.cn (D.X.); zxy1011@whu.edu.cn (X.Z.); 2019203030002@whu.edu.cn (J.P.); 2 Clinical Research Center of Hubei Critical Care Medicine, Wuhan 430071, China; 3 Intensive Care Unit, The Second Affiliated Hospital of Hainan Medical College, Haikou 570100, China

**Keywords:** AKI, CKD, transition mechanisms, targeted therapeutic, maladaptive repair

## Abstract

Acute kidney injury (AKI) causes damage to the renal epithelium, initiating a reparative process intended to restore renal function. Although effective repair can result in the complete recovery of kidney function, this process is frequently incomplete. In instances where repair is unsuccessful, the kidney experiences maladaptive alterations that may progressively result in chronic kidney disease (CKD), a phenomenon referred to as failed repair. This condition is precipitated by hypotensive, septic, or toxic insults, which initiate a series of pathophysiological processes, including microcirculatory dysfunction, the activation of inflammatory responses, and the death of tubular epithelial cells. These events collectively compromise renal function and trigger a complex repair response. This review provides a comprehensive examination of the multifactorial mechanisms underlying the initiation and progression of AKI, the regenerative pathways facilitating structural recovery in severely damaged kidneys, and the critical transition from adaptive repair to maladaptive remodeling. Central to this transition are mechanisms such as epigenetic reprogramming, G2/M cell-cycle arrest, cellular senescence, mitochondrial dysfunction, metabolism reprogramming, and cell death, which collectively drive the progression of CKD. These mechanistic insights offer a robust foundation for the development of targeted therapeutic strategies aimed at enhancing adaptive renal repair.

## 1. Introduction

CKD affects approximately 10% of the global population and frequently progresses to kidney failure [1]. There are no available therapies to reverse its progression, and this leads to significant morbidity and mortality. A common pathological feature across all forms of CKD, regardless of the etiology, is the impairment of epithelial repair, which is increasingly recognized as a significant contributor to interstitial fibrosis [2]. Understanding the initial cellular events that trigger kidney fibrogenesis will enhance our comprehension of CKD pathophysiology and may reveal new and effective therapeutic targets. AKI is characterized by a rapid loss of renal function [3]. Recent clinical and experimental studies have demonstrated a significant association between AKI and CKD, previously regarded as separate conditions [4,5,6]. AKI can contribute to CKD onset and progression, while pre-existing CKD may increase susceptibility to AKI. This review aims to summarize the interrelationship between AKI and CKD and examine the potential mechanisms that contribute to AKI-CKD transition. Of note, epithelial–esenchymal transition (EMT) is also a key mechanism in AKI-CKD progression, but its detailed discussion is beyond the scope of this review.

## 2. Renal Tubular Regeneration: Balancing Adaptive Repair and Maladaptive Responses

The mechanisms underlying renal tubular regeneration following injury have been progressively elucidated through ongoing scientific exploration, particularly regarding whether it is facilitated by surviving mature cells or renal stem cell proliferation. In 2008, researchers demonstrated that surviving renal tubular epithelial cells are the primary source for the repair after AKI [7]. Researchers utilized DNA-labeling techniques to trace cellular processes and found that following acute kidney injury (AKI), proximal tubular epithelial cells (PTCs) in the mouse kidney undergo random dedifferentiation, proliferation, and subsequent redifferentiation into mature PTCs to facilitate tissue repair [8]. Subsequent investigations demonstrated that damaged PTCs initiate proliferation post-AKI, with the majority of the repair process being completed by day 14. However, approximately 20% of PTCs remain in a compromised and dedifferentiated state at this juncture [9]. The damaged proximal tubular cells (PTCs) demonstrate the expression of specific injury-associated genes, such as Havcr1 (Kidney Injury Molecule 1, KIM1), Krt8 (Keratin 8), Krt20 (Keratin 20), and Lcn2 (neutrophil gelatinase-associated lipocalin, NGAL) [10]. Previous research has shown that following acute kidney injury (AKI), the injured proximal tubular cells (PTCs) in a compromised state nearly vanish within two days. By the fourteenth day post-injury, a novel and distinct cell population emerges. These cells undergo differentiation and proliferation, constituting approximately 30% of the PTC population. Notably, around 8% of these cells persist even six weeks post-injury. This observation suggests that this process may play a crucial role in kidney repair and regeneration [11]. In the aforementioned study, the newly identified cell type demonstrated a unique gene expression profile absent in healthy or early-stage acute kidney injury (AKI) proximal tubular cells (PTCs) in mice. This gene set includes markers such as Vcam1, Dcdc2a, and Sema5a, which are considered indicative of the injury repair process. At later time points, PTCs exhibited the downregulation of terminal differentiation marker genes, suggesting an impairment in the differentiation process of PTCs. Consequently, researchers classified these cells as failed-repair proximal tubular cells (FR-PTCs) [11,12]. Subsequent research has corroborated that injury results in the persistent presence of FR-PTCs, marked by Vcam1^+^/Ccl2^+^ expression. These cells display a senescence-associated secretory phenotype, distinguished by the upregulation of proinflammatory and profibrotic gene expression, which contributes to the progression from acute kidney injury (AKI) to chronic kidney disease (CKD) [13]. In a murine model of acute kidney injury (AKI) induced by ischemia–reperfusion injury (IRI), single-nucleus RNA sequencing analysis demonstrated that failed-repair proximal tubular cells (FR-PTCs) are marked by a downregulation of genes associated with normal differentiation and proliferation, such as Hnf4a, alongside an upregulation of genes related to injury, including Havcr1, Krt20, Ccl2, and Vcam1. Among these, Vcam1 showed the most pronounced increase [14]. The majority of FR-PTCs were isolated from samples collected four weeks post-injury. Nevertheless, Vcam1-positive PTCs, which serve as injury markers, were still detectable six months following acute kidney injury (AKI). This persistence suggests their potential role in the transition from AKI to chronic kidney disease (CKD) [15].

Two studies published in 2015 and 2016 examined the role of SOX9, a transcription factor regulating cell proliferation and differentiation during kidney repair, following AKI [16,17]. The 2015 study examined the transcriptome of PTCs and identified that Sox9 expression was rapidly upregulated within 4 h post-injury, peaking at 24–48 h. Compared to sham-operated kidneys, SOX9 expression increased 20-fold. Immunofluorescence analysis demonstrated that in uninjured kidneys, SOX9-positive (SOX9^+^) proximal tubular cells (PTCs) were exceedingly rare, comprising only 0.05% of the proximal tubular cell population, while SOX9^+^ distal tubular cells were also limited in number. Following acute kidney injury (AKI), 10.6% of proximal tubular cells expressed SOX9. A majority of these SOX9^+^ cells coexpressed the dedifferentiation marker KIM-1, with 40% of them actively proliferating. Previous studies have demonstrated that following AKI, SOX9^+^ PTCs re-enter mitosis and significantly promote the extensive repair of the injured proximal tubules [16,17]. A recent study by Sanjeev Kumar et al. demonstrated the critical role of SOX9 expression after AKI in renal tissue repair [18]. The research revealed that SOX9^+^ cells continued to proliferate on the 10th day post-AKI. The expression level of SOX9 was found to be downregulated; however, partially recovered proximal tubular cells (PTCs) continued to express SOX9. This persistent expression of SOX9 led to renal interstitial fibrosis and tissue scarring. Importantly, the researchers identified that inhibiting SOX9 activity could hinder the progression of renal fibrosis. SOX9-positive cells were associated with the expression of fibrotic markers. Furthermore, the research team demonstrated that SOX9-positive PTCs secrete Wnt4, a key factor in sustaining Wnt-β-catenin activity within the profibrotic fibroblast microenvironment. Studies involving human kidney transplant biopsy specimens support these findings, revealing a positive correlation between SOX9 expression and the severity of renal interstitial fibrosis. Moreover, SOX9 expression was correlated with cadherin 6 (CDH6), Wnt2b, and the expression of profibrotic genes [18]. These findings underscore the critical importance of sustained SOX9 expression in the progression of renal fibrosis following acute kidney injury (AKI). They contribute to a deeper understanding of the mechanisms underlying kidney repair and identify novel molecular targets for future therapeutic interventions against chronic kidney disease (CKD). Notably, the expression of SOX9 in human transplant kidney biopsy specimens shows a positive correlation with the extent of fibrosis, highlighting its potential as a therapeutic target. Future research should concentrate on devising spatiotemporally specific regulatory strategies to reconcile the dual roles of SOX9 in early repair processes and late-stage fibrosis.

During acute kidney injury (AKI), injured proximal tubular cells (PTCs) can undergo processes of dedifferentiation, proliferation, and subsequent redifferentiation, collectively termed adaptive repair, to facilitate endogenous recovery. PTCs that successfully engage in adaptive repair contribute to renal recovery post-AKI. However, in instances of severe injury, PTCs may be unable to repair effectively due to disruptions in energy metabolism or DNA damage, resulting in what is termed “maladaptive repair”. PTCs undergoing maladaptive repair are not merely passive victims of AKI; rather, they exhibit distinct characteristics such as cell-cycle arrest, apoptosis, metabolic dysfunction, senescence, and sustained inflammatory responses. These features culminate in tubular atrophy and degeneration, interstitial fibrosis, and a decline in renal function, thereby advancing the transition from AKI to chronic kidney disease (CKD). While adaptive repair mechanisms can effectively restore tubular architecture following mild to moderate injury, severe or recurrent insults may lead to dysregulated repair processes.

## 3. Mechanisms of Maladaptive Repair

### 3.1. Epigenetic Reprogramming

Emerging evidence suggests that epigenetic regulation is pivotal in determining cell fate by orchestrating temporal and spatial regulation [19,20]. Chromatin accessibility, which refers to the degree of chromatin compaction, is a fundamental characteristic of chromatin states and is widely acknowledged as a hallmark of active cisregulatory elements [21,22]. Regions of accessible chromatin, characterized by reduced nucleosome presence and diminished chromatin compaction, serve as sites for the recruitment of transcription factors (TFs) through DNA-specific interactions. The binding of TFs, in turn, plays a crucial role in establishing and maintaining the openness of these regulatory regions. Based on the sequence characteristics of accessible chromatin regions, TF binding can be predicted, allowing for the mapping of dynamic TF regulatory networks. Consequently, analyzing chromatin dynamics and profiling TF regulatory networks following AKI may illuminate the epigenetic mechanisms underlying cellular responses to varying degrees of injury, thereby identifying key regulators involved in both adaptive and maladaptive kidney repair.

In related studies, researchers have systematically defined cell type–specific epigenetic signatures in healthy kidneys [23] as well as in kidneys from patients with autosomal dominant polycystic kidney disease and CKD [24,25]. These investigations revealed that FR-PTCs exhibit a significant enrichment of nuclear factor κB (NF-κB) transcription factor binding sites within accessible chromatin regions. This increased accessibility of NF-κB motifs in FR-PTCs suggests a central role for this proinflammatory transcription factor in driving inflammation, fibrosis, and the AKI-CKD.

Recent research by Muto et al. delineates the dynamic epigenetic landscape underlying the transition from AKI-CKD [14]. Utilizing single-nucleus multiomic profiling of approximately 280,000 nuclei throughout a mouse AKI time course, and corroborating findings in human AKI samples, the research demonstrates that fibroblast-like proximal tubular cells (FR-PTCs) exhibit enduring changes in chromatin accessibility, predominantly influenced by NF-κB transcription factor activity. NF-κB orchestrates proinflammatory and profibrotic gene expression programs through conserved cisregulatory elements (CREs), including chemokines such as CCL2 and CSF1, and anti-phagocytic signals like CD47, which collectively recruit immune cell subsets and maintain fibrotic microenvironments. Cross-species regulatory network analysis identified CREB5 as a bifunctional regulator, facilitating tubular proliferation during acute repair phases while contributing to maladaptive FR-PTC states in chronic phases. Knockdown of CREB5 resulted in suppressed proliferation via activation of the p53 pathway, underscoring its context-dependent roles. Additionally, immune cell profiling revealed macrophage heterogeneity, including CCR2+ M1 and CSF1R+ subsets. This study pioneers an integrative methodological approach by combining single-nucleus assay for transposase-accessible chromatin sequencing (snATAC-seq), single-nucleus RNA sequencing (snRNA-seq), and cleavage under targets and release using nuclease (CUT&RUN) to map temporal chromatin dynamics and validate conserved transcription factors.

### 3.2. Cellular Senescence and G2/M Cell-Cycle Arrest

Maladaptive repair of PTCs may occur due to the inability to complete normal mitosis, resulting in cells entering a state of cell-cycle arrest or senescence. Cellular senescence is an irreversible state of cell-cycle arrest induced by various injurious factors. These factors result in upregulating the tumor suppressor gene P53, which subsequently activates downstream P21CIP1 (P21) expression. This, subsequently, inhibits multiple cyclin expressions, resulting in cell-cycle arrest. Moreover, P16INK4A/pRb (P16), which inhibits cyclin-dependent kinases (CDKs), including CDK4/6, is upregulated, stabilizing cells in a cycle arrest state [26]. In contrast to quiescent and fully differentiated cells, senescent cells cannot re-enter the cell cycle in response to stimuli. Senescent cells exhibit specific changes in morphology, metabolism, gene expression, and chromatin remodeling and develop a proinflammatory phenotype [27,28]. In a mouse model of nephrotoxicity caused by repeated low-dose intraperitoneal cisplatin injection, PTCs underwent senescence, which contributed to the AKI-CKD progression. Pharmacological clearance of senescent PTCs reduced renal inflammation, mitigated renal interstitial fibrosis, and enhanced kidney function [29]. These findings suggest that the senescence of PTCs following injury is an essential mechanism in the AKI-CKD progression. In kidney samples from clinical patients with CKD, PTCs senescence persisted despite the alleviation of ureteral obstruction. Furthermore, the degree of cellular senescence correlated with the severity of fibrosis [30]. Renal scRNA-seq analysis revealed that senescent PTCs can facilitate AKI-CKD progression by activating mesenchymal fibroblasts, resulting in renal interstitial fibrosis [31].

Cell-cycle checkpoints involve cyclins, CDKs, and cyclin-dependent kinase inhibitors (CKIs), which inhibit cell division in response to DNA damage or cellular stress. These checkpoints safe-guard cellular energy by inhibiting unnecessary progression through the cell cycle during adverse conditions [32]. Previous studies have demonstrated that G2/M cell-cycle arrest significantly contributes to maladaptive repair after AKI and AKI-to-CKD progression. In various AKI models, including bilateral IRI, unilateral IRI, aristolochic acid-induced injury, and unilateral ureteral obstruction (UUO), injured PTCs exhibit G2/M phase arrest. This is accompanied by the synthesis of profibrotic growth factors, which contribute to the progression of renal fibrosis [33]. The pharmacological inhibition of cells in G2/M cell-cycle arrest diminishes renal interstitial fibrosis. Conversely, an increased proportion of cells arrested in the G2/M phase exacerbates renal interstitial fibrosis [34].

### 3.3. Mitochondrial Dysfunction and Metabolism Reprogramming

#### 3.3.1. Mitochondrial Damage

Acute kidney injury (AKI) can lead to mitochondrial dysfunction, predominantly as a consequence of hypoxia interfering with the electron transport chain (ETC) within mitochondria. This interference results in the overproduction of reactive oxygen species (ROS), which are detrimental free radicals. The excessive ROS exacerbate damage to renal tubular cells, thereby contributing to the progression of injury [35]. Recent studies have demonstrated that mitochondrial dysfunction is a key factor driving AKI-to-CKD progression. This dysfunction hinders energy production, enhances oxidative stress, and initiates maladaptive repair mechanisms, leading to chronic kidney injury and fibrosis [35]. Mitochondrial homeostasis is maintained through mitochondrial dynamics, mitophagy, and mitochondrial biogenesis. Mitochondrial dynamics involve two distinct processes: fission, regulated by DRP1 (Dynamin-Related Protein 1), and fusion, regulated by MFN1 (Mitofusin 1), MFN2 (Mitofusin 2), and OPA1 (Optic Atrophy 1) [36,37]. Mitophagy refers to the selective degradation of damaged mitochondria via autophagy. This process is regulated by several pathways, including the PINK1–PARK2 pathway, the BNIP3 and NIX pathways, and the FUNDC1 pathway. Additionally, mitochondrial biogenesis, which is primarily governed by peroxisome proliferator-activated receptor gamma coactivator-1α (PGC-1α), fulfills the high energy demands of the cell and replenishes mitochondrial content during cellular proliferation [38,39]. Animal studies have demonstrated that regulating the genes associated with mitochondrial homeostasis influences renal inflammation, kidney fibrosis, and apoptosis [40]. Increased levels of PGC-1α in renal tubular cells enhance mitochondrial mass and ameliorate kidney injury [41]. Conversely, PGC-1α deficiency in a sepsis-induced AKI model results in severe renal dysfunction [42]. Studies have demonstrated that improving mitochondrial dynamics by inhibiting Drp1-dependent fission, preserving mitochondrial membrane potential, and regulating mitophagy can mitigate mitochondrial dysfunction. Accordingly, regulating mitochondrial dynamics may offer new therapeutic avenues for AKI. Several studies have demonstrated that autophagy acts as a protective mechanism by eliminating excessively produced collagen, reducing oxidative stress, and inhibiting endothelial-to-mesenchymal transition, thus preventing renal interstitial fibrosis after AKI [43]. However, some studies indicate that the prolonged activation of autophagy during renal repair can induce a profibrotic phenotype in tubular cells, including senescence and the secretion of proinflammatory and profibrotic cytokines. Therefore, the role and regulatory mechanisms of autophagy in maladaptive renal repair necessitate additional investigation.

#### 3.3.2. Metabolism Reprogramming

PTCs constitute approximately 50% of the total cellular population in the kidney and exhibit a significant energetic demand necessary for maintaining acid–base homeostasis, as well as for the reabsorption and secretion of water, ions, and other compounds [44]. PTCs predominantly depend on mitochondrial fatty acid oxidation (FAO) to generate the requisite adenosine triphosphate (ATP). In contrast to cortical tubule segments (specifically, the S1 and S2 segments), medullary (S3) segments exhibit a greater reliance on glycolysis rather than oxidative phosphorylation (OXPHOS), attributed to the reduced partial pressure of oxygen (pO2) in the medulla compared to in the cortex [45]. FAO is instrumental in mediating the response of proximal tubular epithelial cells to injury, given that lipids provide an essential energy source for the regeneration of proliferative tubules. During the initial phase of ischemia, FAO is temporarily upregulated in murine proximal tubular cells, a phenomenon that aligns with successful cellular repair in vivo. This observation has been substantiated by analyses of human datasets [46]. Conversely, the impairment of FAO is a pathogenic characteristic of CKD. Patients with CKD exhibit a significant reduction in FAO and OXPHOS, which is evidenced by the accumulation of intracellular lipids and the diminished expression of key transcription factors associated with mitochondrial metabolism [47,48]. These transcription factors include estrogen-related receptor α and PGC1α, which are crucial for mitochondrial biogenesis and fatty acid metabolism; mitochondrial transcription factor A (TFAM), which is essential for mitochondrial DNA transcription and replication; as well as peroxisome proliferator-activated receptor α (PPARα) and Krüppel-like factor 15 (KLF15), which are involved in the regulation of fatty acid catabolism [49,50,51]. Several mechanisms govern the suppression of fatty acid oxidation (FAO) associated with chronic kidney disease (CKD) in proximal tubular cells (PTCs). Transforming growth factor-beta (TGFβ)-induced SMAD3 is pivotal in this process, as it inhibits the expression of genes responsible for fatty acid uptake and oxidation. This inhibition occurs through SMAD3’s binding to the promoter region of Ppargc1a and the subsequent suppression of histone 3 lysine 4 monomethylation (H3K4Me1). The impairment of FAO in PTCs promotes their transition to a secretory phenotype, resulting in the increased expression of inflammatory cytokines such as interleukin-1 beta (IL-1β), interleukin-6 (IL-6), and tumor necrosis factor (TNF), which in turn attract macrophages to the site of injury. Importantly, a study published in 2024 revealed that the conditional deletion of Cpt1a in proximal tubular epithelial cells (PTECs) in vivo led to enhanced macrophage infiltration, while exerting a minimal effect on kidney injury and fibrosis [52]. This observation suggests that peroxisomal FAO may compensate for the adverse effects associated with Cpt1a deletion and impaired mitochondrial FAO. Furthermore, this finding underscores the role of FAO in the pathogenesis of CKD by influencing both cellular energy metabolism and the accumulation of toxic lipids.

Lipid droplets are present in almost all damaged renal parenchymal cells. A 2015 large-scale genome-wide transcriptome analysis revealed that inflammation and metabolism are the most dysregulated pathways in diseased human kidneys [49]. The study found that in human and mice fibrotic kidneys, the levels of key enzymes and regulators associated with FAO in PTCs were diminished, accompanied by lipid accumulation. Mice exhibiting tubular-specific overexpression of the long-chain fatty acid transporter CD36 predominantly deposited fatty acid, stearic acid, palmitic acid, linoleic acid, and docosahexaenoic acid in the kidney [53]. Significant lipid accumulation in PTCs has been observed in angiotensin II-induced rats, high-fat-diet-fed mice, and cisplatin-induced nephrotoxicity models [54,55].

**Figure 1:**
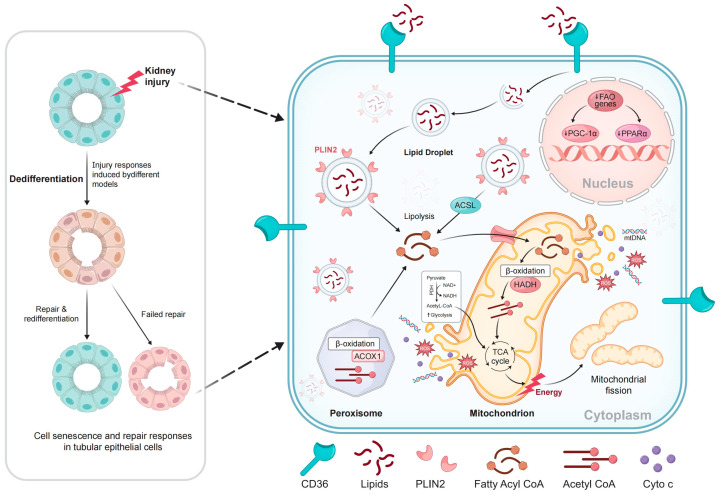
Proposed model of activated lipid metabolism in injured proximal tubule cells: CD36 functions as a transporter for long-chain fatty acids. Within the cell, these fatty acids accumulate and form lipid droplets, with PLIN2 serving as a surface protein. Fatty acyl-coenzyme A (CoA) is produced from lipids through ACSL-mediated lipolysis or lipophagy and is utilized in ACOX1-mediated peroxisomal β-oxidation or CPT-mediated mitochondrial β-oxidation. This process generates acetyl-CoA, a key substrate for TCA, which produces energy. CPT1 and CPT2, which transport Acyl-CoA into mitochondria, contribute to decreased FAO, ineffective NADH production, reduced ETC activity, and inadequate ATP levels, ultimately leading to mitochondrial dysfunction. Furthermore, data from animal models indicate that the suppression of genes involved in mitochondrial biogenesis, particularly PGC-1α and PPARα, leads to mitochondrial DNA instability.

A previous study has demonstrated that prolonged fatty acid uptake (10-day palmitic acid treatment) stimulates inflammatory and profibrotic mechanisms in mouse renal PTCs [56]. Recent research demonstrated that injured PTCs exhibit a transient lipid accumulation and a significant increase in FAO-related gene expression within 6 h post-injury [12]. Subsequent analysis using the bulk RNA sequencing of cells treated with oleic acid for 6 h and collected on day 2 demonstrated the upregulation of genes involved in DNA replication, the cell cycle, and proliferation, signifying a repair state with high energy demands. Similarly, in vivo experiments revealed that PTCs expressing the repair marker Mki67 were most prevalent in the kidneys of IRI mice on the second day after injury. These findings indicate that lipid deposition within the first 6 h following kidney injury may serve as a vital energy source for damaged epithelial cells. The proliferation and expansion of PTCs facilitate tubular repair during this phase [12]. CD36 functions as a transporter for long-chain fatty acids, which subsequently accumulate within the cell and form lipid droplets, with PLIN2 acting as a surface protein. Fatty acyl-coenzyme A (CoA) is synthesized from lipids through ACSL-mediated lipolysis or lipophagy and is utilized in ACOX1-mediated peroxisomal β-oxidation or CPT-mediated mitochondrial β-oxidation. This metabolic pathway generates acetyl-CoA, a critical substrate for the tricarboxylic acid (TCA) cycle, which is essential for energy production. The transport of acyl-CoA into mitochondria via CPT1 and CPT2 is associated with reduced fatty acid oxidation (FAO), inefficient NADH production, decreased electron transport chain (ETC) activity, and insufficient ATP levels, ultimately leading to mitochondrial dysfunction. Furthermore, evidence from animal models indicates that the inhibition of genes involved in mitochondrial biogenesis, particularly PGC-1α and PPARα, results in mitochondrial DNA instability (Figure 1).

### 3.4. Programmed Cell Death

Mitochondrial damage can precipitate various forms of cell death, including apoptosis, necroptosis, pyroptosis, and ferroptosis, also promoting the release of the proinflammatory cytokines that facilitate immune cell infiltration and fibroblast activation. However, further research is necessary to elucidate the intricate relationships between metabolic processes, various forms of cell death, and fibrotic responses within the renal microenvironment.

#### 3.4.1. Ferroptosis

Ferroptosis is an iron-dependent form of cell death driven by lipid peroxidation. Through the creation of a mouse UUO-induced renal fibrosis model, researchers observed that PTCs exhibited the typical features of ferroptosis concurrent with the development of fibrotic pathological phenotypes in the kidneys. The inhibition of ferroptosis via liproxstatin-1 diminished the expression of profibrotic factors and the activation of myofibroblasts in the UUO model, consequently alleviating renal interstitial fibrosis [57]. Subsequent research employing diverse models of kidney injury and repair, in conjunction with single-cell RNA sequencing (scRNA-seq) and murine genetic techniques, has elucidated that proximal tubular cells (PTCs) adopt a distinctive proinflammatory phenotype and undergo ferroptosis following injury [10]. Although these inflammatory PTCs manifest transiently and revert to normal after mild injury, their accumulation following severe kidney injury results in chronic inflammation and renal interstitial fibrosis. Furthermore, the transient inflammatory PTC state is marked by the significant downregulation of glutathione metabolism genes, making the cells more susceptible to ferroptosis. Mild injury-induced ferroptosis inhibits the redifferentiation of injured PTCs into normal PTCs, resulting in the accumulation and persistence of inflammatory PTCs. This promotes maladaptive repair and leads to renal interstitial fibrosis. The study demonstrated that the pharmacological inhibition of ferroptosis significantly alleviates inflammation and diminishes renal interstitial fibrosis after AKI, affirming that ferroptosis is a crucial mechanism regulating the repair and regeneration of PTCs following kidney injury [10].

#### 3.4.2. Pyroptosis

Pyroptosis is a form of inflammatory cell death marked by cellular swelling and the secretion of extracellular vesicles from the plasma membrane. Gasdermin D (GSDMD), a substrate for all inflammatory caspases, is essential in executing inflammasome-induced cell death, thus facilitating pyroptosis [58]. Recent observations have identified pyroptosis as a significant process in various kidney diseases. The NLRP3 inflammasome, a pivotal component in the pyroptotic pathway, plays a critical role in modulating innate immune responses by regulating the maturation and secretion of proinflammatory cytokines, such as interleukin-1β (IL-1β) [59].

Researchers have detected the activation of Casp3 and the cleavage of the GSDME protein in a mouse UUO-induced renal fibrosis model, and knockout of GSDME or Casp3 inhibited tubular cell pyroptosis, thereby alleviating hydronephrosis, interstitial fibrosis, and inflammatory cell infiltration [60]. Meanwhile recent single-cell data analysis revealed that ferroptosis and pyroptosis in PTCs induce cell death and exacerbate cellular damage, leading to tubular repair failure and facilitating AKI-to-CKD progression [61]. The findings demonstrated the persistent enrichment of cell death-related genes (pyroptosis and ferroptosis) in injured PTCs, strongly associated with maladaptive repair in AKI-CKD progression. The pharmacological inhibition of pyroptosis and ferroptosis enhanced maladaptive renal repair and interstitial fibrosis after severe IRI, thereby mitigating AKI-to-CKD progression [61,62].

These studies highlight the critical roles of pyroptosis and ferroptosis in facilitating renal fibrosis and hindering post-injury repair. The findings enhance our understanding of the mechanisms involved in kidney injury and repair and provide potential therapeutic targets for preventing and treating AKI-CKD progression.

#### 3.4.3. Necroptosis

Necroptosis, a regulated lytic form of cell death, is characterized by the specific formation of a necrosome comprising receptor-interacting protein kinase (RIPK) 1, RIPK3, and the mixed lineage kinase domain-like protein (MLKL) [63,64]. Our previous research demonstrated that the necroptosis of tubular cells, induced by cisplatin nephrotoxicity, initiates necroinflammation [65]. The inhibition of RIPK1 (via Nec-1) [66] and the deletion of RIPK3 or MLKL mitigates renal dysfunction following IRI [67,68,69,70]. Moreover, the deletion of caspase 8 leads to severe chronic inflammation; however, the simultaneous deletion of RIPK3 and caspase 8 inhibits this inflammatory response [71]. These findings highlight the role of necrosome components in modulating inflammatory reactions.

Studies demonstrate that RIPK3-MLKL necroptotic signaling activates the NLRP3 inflammasome when caspase 8 is suppressed. Caspase 8 activation in the kidney was effectively inhibited after IRI [72]. Mice lacking Ripk3 or MLKL demonstrated decreased NLRP3 inflammasome activation after a similar initial kidney injury as observed in WT mice (35 min ischemia), indicating that RIPK3 and MLKL are involved in inflammasome activation. The study clarified that RIPK3-MLKL-dependent necroptosis triggers NLRP3 inflammasome activation in an auto-amplification loop, which plays a significant role in AKI-to-CKD progression [73].

## 4. Signaling Pathways During the Renal Tubular Repair Process

Recent studies have demonstrated that the upregulation of Notch, Wnt, and Hedgehog (Hh) signaling pathways in renal tubular epithelial cells is a key event that drives their differentiation, a process critically linked to the pathogenesis of CKD [74,75,76]. The augmented signaling induces phenotypic alterations in proximal tubular cells (PTCs), disrupting normal cellular function and diminishing the regenerative capacity of the renal epithelium. This, in turn, contributes to maladaptive repair mechanisms and fibrosis. Moreover, the interaction among these signaling pathways collectively influences the processes of kidney injury and repair [77,78]. Understanding these intricate interactions provides valuable insights into the molecular underpinnings of CKD and highlights potential therapeutic targets.

### 4.1. Wnt

The Wnt pathway is a universally present and highly conserved signal transduction pathway in organisms, essential for numerous physiological processes, including embryonic development, cell proliferation, differentiation, migration, and maintenance of tissue homeostasis [79]. Wnt proteins are secreted glycoproteins that function by binding to the Frizzled family receptors and co-receptors of low-density-lipoprotein receptor-related protein 5/6 [80]; they recruit and activate the intracellular disheveled protein near the cell membrane, subsequently exerting physiological effects in a β-catenin-dependent or -independent manner [81]. After β-catenin translocation into the nucleus and its binding to T-cell factor/lymphoid enhancer factor (TCF/LEF) transcription factors, it activates downstream pathways, including TGF-β1/SMAD signaling, Snail1, and Twist1 [82].

Wnt expression at the transcriptional and translational levels significantly increased in the IRI [83] and was closely associated with severity [84]. Pretreatment with a Wnt agonist [85] and exogenous Wnt 1 h before ischemia may mitigate kidney injury [86], while tubular-specific knockout of β-catenin exacerbated ischemia-induced kidney injury and nephrotoxicity [87]. Wnt4 or β-catenin primarily protects PTCs from apoptosis and mitigates AKI kidney injury by enhancing cell proliferation and inhibiting the activation of p53 and Bax [88,89]. Additionally, Wnt is implicated in cellular communication in the kidney. The Wnt7 interaction between macrophages and renal tubular cells is essential in kidney injury [90]. The intercellular communication of Wnt between proximal renal tubules and activated perivascular fibroblasts/pericytes promotes interstitial fibrosis [91,92], and extrarenal myeloid-derived Wnt is essential for the activation of macrophages and fibroblasts contributing to renal fibrosis [93,94]. The function of Wnt/β-catenin in kidney injury repair is complex. In several chronic kidney injury conditions, including UUO [95] and nephropathy [96], the upregulation of the Wnt pathway has also been observed, and this upregulation appears to exacerbate chronic kidney injury. Transgenic mice exhibiting activated β-catenin have been demonstrated to spontaneously develop renal lesions and fibrosis [97]. In chronic kidney injury models, administering various antagonists (including secreted frizzled-related protein 4, Klotho, and the small molecule inhibitor ICG-001) to inhibit the Wnt/β-catenin signal has resulted in a reduction in renal fibrosis and the alleviation of proteinuria [98]. In CKDs, Wnt is typically considered harmful; however, some studies have identified that the interaction between tubular β-catenin and FoxO3 may confer a protective effect in CKD [99].

In addition to the previously mentioned pharmacological inhibitory effects, recent studies indicate that some endogenous small molecules, proteins, and RNAs exacerbate chronic kidney injury by upregulating the Wnt pathway, while pregnane X receptor, combined with melatonin and poricoic acid A, exhibits the opposite effect. Previous studies have demonstrated that the classical Wnt pathway, specifically the β-catenin-dependent pathway, is essential in the bidirectional role in kidney injury repair. It mitigates kidney injury during the acute phase; however, continuous activation can cause CKD progression (Table 1).

### 4.2. Notch

The Notch pathway, similar to the Wnt pathway, is a highly conserved intercellular signal transduction pathway that is essential for various physiological processes, including cell proliferation, differentiation, and apoptosis [113]. The Notch signaling pathway is fundamentally constituted by Notch receptors, ligands, and intracellular effector molecules. In mammals, there are four distinct Notch receptors (Notch1–4), with ligands predominantly belonging to the Delta-like (Dll1, Dll3, Dll4) and Jagged (Jag1, Jag2) families. Upon ligand–receptor interaction, the Notch receptor undergoes enzymatic cleavage, resulting in the release of the Notch intracellular domain. This domain then translocates to the nucleus, where it associates with transcription factors to activate downstream target genes [114], thus regulating the physiological functions of cells.

In various kidney injury models, the ligands and receptors associated with Notch signal transduction are upregulated [115,116], and the degree of fibrosis is positively correlated with Notch levels. Furthermore, the expression of Notch pathway proteins correlates with albuminuria, glomerulosclerosis, and renal function [117], while inhibiting the Notch pathway mitigates kidney injury [118]. Notch is extensively distributed. While its expression level is low in healthy organisms, the expression levels of various cells, including myeloid-derived cells, podocytes, epithelial cells, and endothelial cells, are significantly increased in chronic kidneys. Additionally, the reactivation of Notch signal transduction in the kidneys of animal models and patients with CKD has been demonstrated to result in epithelial damage and the progression of fibrosis. Inhibiting the Notch pathway can mitigate renal fibrosis progression [74]. Activated Notch inhibits the expression of the adaptor protein Disabled-2 in tubular epithelial cells, thereby protecting them from TGF-β-induced epithelial-to-mesenchymal transition and IRI [119]. The overexpression of active Notch1 in tubular epithelial cells aggravated renal fibrosis, while the inhibition of Notch resulted in the amelioration of renal fibrosis [120]. The intercellular signal crosstalk of Notch significantly contributes to kidney injury repair. Notch signal transduction induces renal fibrosis through endothelial-to-mesenchymal transition surrounding the renal tubules [121]. In Diabetic Nephropathy (DN), the Notch signal transduction of podocytes is related to the degree of glomerulosclerosis [117]. Furthermore, previous studies have found that the Notch pathway in bone marrow-derived cells is essential in chronic kidney injury. In DN, the Notch pathway in macrophages promotes macrophage polarization, exacerbating the inflammatory reaction, fibrosis, and necroptosis of the kidneys. Inhibiting the Notch pathway in macrophages alleviates the pathological changes in renal cells [122]. Moreover, in the UUO model, myeloid-specific targeting of Notch ameliorates renal fibrosis in mice by diminishing the infiltration and activation of bone marrow-derived macrophages [123]. Comprehensive studies have revealed that specifically blocking Notch in ferroptosis suppressor protein 1-positive cells derived from bone marrow can improve renal fibrosis in UUO mice [124]. While substantial research evidence suggests that the Notch pathway exacerbates renal fibrosis and CKD progression, it serves as a double-edged sword in AKI. The Notch pathway is involved in the repair process of gentamicin-induced AKI [118,125], while Notch 3 aggravates the IRI [126] (Table 2).

### 4.3. Hedgehog

The Hedgehog signaling pathway primarily comprises ligands, receptors, and intracellular signaling molecules with transcription factors. The Hedgehog ligands comprise Sonic Hedgehog (Shh), Indian Hedgehog (Ihh), and Desert Hedgehog (Dhh). The primary transmembrane receptors comprise patched (Ptch) and smoothened (Smo). With no Hh signal, Ptch inhibits Smo activity. After the binding of the Hh protein to Ptch, the inhibitory effect of Ptch on Smo is alleviated. Activated Smo can transmit the signal to the Gli family proteins of mammalian transcription factors through intracellular signaling molecules. Gli proteins subsequently translocate to the nucleus to initiate the transcription of downstream target genes [134].

The Shh signal has been widely studied in kidney injury. Activating Shh in the early stage of AKI mitigates AKI induced by IR and sepsis, which may be linked to the fact that the early Shh pathway helps cell proliferation and reduces cell apoptosis [135,136]. During CKD, the Hedgehog signaling pathway is essential in renal fibrosis development, primarily through the secretion of Hh ligands by epithelial cells and the upregulation of Gli1 in myofibroblasts [137]. Shh expression in the tubular epithelial cells of mouse models of renal fibrosis induced by UUO and IRI is elevated, and the inhibition of the Shh signaling pathway has a protective effect in various kidney injury models, including hypertension [138], diabetes [139], UUO [140], and ciaplatin [140]. Cyclopmine is an effective SMO inhibitor, and its inhibition of the Hh signal significantly alleviates the progression of renal fibrosis in mouse models of IRI and obstructive nephropathy [141]. The genetic ablation of Gli1 reduces kidney injury in mouse models of renal fibrosis [142]. The roles of other Hh proteins in kidney injury repair are being gradually investigated. Ihh release from tumor necrosis factor-activated renal epithelia drives renal fibrosis [143,144]. The Hh signal plays an important role in intercellular communication. The Shh ligand shuttled by exosomes derived from renal tubules plays an essential role in renal fibrosis [145].

Notch, Wnt, and Hedgehog signaling pathways are essential for embryonic development, cellular proliferation, differentiation, and migration. Their expression levels are higher and essential in kidney injury repair. In the early stage of AKI, these characteristics promote cell proliferation and reduce cell death, partially alleviating kidney injury. However, continuous overactivation exacerbates epithelial–mesenchymal transition and renal fibrosis, thereby promoting the transformation of AKI-CKD (Table 3).

## 5. Emerging Therapeutic Strategies and Future Directions

### 5.1. Exosomes in Kidney Injury and Repair

Exosomes (Exos), which are a heterogeneous group of double-membrane-bound vesicles, play a crucial role in mediating intercellular communication by shuttling molecular cargo from donor cells to recipient cells. Exos released from different cells exert distinct effects and can be selectively taken up by neighboring or distant cells, thereby reprogramming the recipient cells based on their bioactive compounds [146]. Researchers found that FRC-Exos protect the kidneys from sepsis-induced injury [147]. Exosomes from multiple sources, particularly those derived from mesenchymal stem cells, have demonstrated a reparative role in various types of AKI by promoting the proliferation and repair of renal tubules, regulating autophagy, inhibiting inflammatory responses and oxidative stress, suppressing fibrosis progression, and facilitating angiogenesis [148]. However, paracrine exosomes in the kidneys are predominantly detrimental, especially in the cellular communication between RTECs and inflammatory cells [149,150]. Injury-induced exosomes micro374b-5p derived from RTECs promote the activation of M1 macrophages [150], while exosomes derived from macrophages cause glomerular endothelial cell dysfunction [151], trigger pyroptosis [152], and participate in the apoptotic injury and inflammatory responses that promote AKI. In the advanced stage of AKI, exosomes significantly contribute to the mechanism of renal fibrosis and repair. The primary source of exosomes for mitigating the AKI-CKD progression derived from renal tubular cells activates fibroblasts and facilitates kidney fibrosis [153].

Current research in the field of extracellular vesicles is focused on enhancing the targeting efficiency of Exos to specific tissue types. Exosomal transport represents a novel mechanism for intercellular communication during injury [154]. Notably, FRC-Exos demonstrate a capacity to specifically target damaged renal tissue, as evidenced by fluorescence observed in PTCs following coculture with FRC-Exos. Additionally, the incorporation of a ligand derived from an LTH-targeting peptide segment, which selectively binds to KIM-1 expressed on injured kidney cells, significantly improves the targeting efficacy of Exos toward kidney tubular cells [154]. These findings suggest that Exos are promising drug-delivery vehicles with great targeted therapeutic potential for AKI-CKD.

### 5.2. Metabolic Interventions

The activation of PPARα has emerged as a promising strategy to restore FAO and counteract lipotoxicity in PTCs. PPARα agonists such as fenofibrate have been shown to upregulate carnitine palmitoyl transferase 1a (CPT1a), thereby improving mitochondrial function and reducing lipid-induced damage [155]. Preclinical studies in various models of kidney injury indicate that fenofibrate not only mitigates renal lipid accumulation but also enhances overall renal function. Moreover, ongoing research into novel, more selective PPARα modulators promises to refine this therapeutic approach by enhancing efficacy while minimizing off-target effects.

Aberrant glycolytic reprogramming is a critical driver of maladaptive repair and fibrosis in the kidney. Small-molecule inhibitors of 6-phosphofructo-2-kinase/fructose-2,6-biphosphatase 3 (PFKFB3), such as 3PO, have been shown to reduce glycolytic flux [156]. By doing so, these inhibitors can reverse the histone acetylation changes that drive fibrotic processes. Preclinical studies indicate that modulating glycolysis may restore metabolic homeostasis in injured renal cells, suggesting that the further optimization of PFKFB3 inhibitors could yield effective antifibrotic therapies.

### 5.3. Clearance of Senescent Cells

The accumulation of senescent cells, characterized by the expression of p16INK4a, contributes to chronic inflammation and the progression of renal interstitial fibrosis. Senolytic agents, such as the combination of dasatinib and quercetin, have demonstrated the capacity to selectively eliminate these p16INK4a-positive cells, thereby attenuating fibrosis and preserving renal function [29]. This approach is supported by emerging clinical data in age-related diseases and holds significant promise for translation into renal therapies.

By targeting core mechanisms, developing innovative technologies, and advancing precision medicine, there is hope for breaking the vicious cycle of AKI-CKD progression in the future. The key challenge lies in balancing efficacy and safety while achieving personalized interventions through the integration of multiomic data. Interdisciplinary collaborations will accelerate the clinical translation of therapeutic strategies. Aging contributes to immunosenescence and may elucidate the increased susceptibility to AKI-CKD progression. Considering the aging population and the escalating burden of age-associated progressive CKD, developing innovative therapies for this widespread disease should be prioritized.

The most prominent current limitation in AKI-CKD transition research lies in the substantial disconnect between basic research and clinical practice. While mechanistic studies have made significant progress in identifying molecular pathways, translational research remains limited in both quantity and depth. Moving forward, extensive clinical studies will be essential to effectively bridge this gap and transform theoretical knowledge into practical therapeutic applications (Table 4). 

## 6. Conclusions

AKI frequently results in considerable morbidity and mortality due to the intricate processes involved in renal damage and subsequent recovery. Post-AKI, renal tubular cells may undergo either adaptive or maladaptive repair pathways. Maladaptive repair is characterized by phenomena such as cellular senescence, cell-cycle arrest, mitochondrial dysfunction, metabolic reprogramming, programmed cell death, and epigenetic reprogramming. Key signaling pathways, including Notch, Wnt, and Hedgehog, play significant roles in these processes. Additionally, exosomes demonstrate a dual role in the context of AKI. A thorough understanding of these mechanisms is crucial for the development of therapeutic strategies aimed at preventing the progression of AKI to CKD. Future research should prioritize the detailed elucidation of these pathways and the identification of therapeutic targets to improve patient outcomes.

## Figures and Tables

**Table 1:** The regulation of the Wnt pathway in kidney injury.

Regulator	In Vivo Models	In Vitro Models	Target or Mechanism	Influence on Kidney	Reference
Fibroblast growth factor 21	UUO	TGF-β	Negative feedback mode	Inhibits renal tubulointers	[100]
Sirtuin 6	UUO, IR	TGF-β	Deacetylation of histone H3K56	Inhibits renal tubulointerstitial fibrosis	[101]
Kallistatin	UUO	TGF-β	Modulation of Wnt4/β-catenin signaling	Inhibition of epithelial–mesenchymal transition	[102]
Transmembrane protein 16 A	Ang II	Ang II	Inhibited the expression Wnt3a, LRP5, and active β-catenin	Inhibits renal tubulointerstitial fibrosis	[103]
Pregnane X receptor	UUO	TGF-β	Interacting with p53 and inhibiting the Wnt7a/β-catenin	Alleviates renal fibrosis	[104]
Combined melatonin and poricoic acid A	IR	HK2: hypoxia–reoxygenation, TGF-β	Disturbed the interaction of Smad3 and β-catenin	Inhibits renal fibrosis	[105]
Claudin-5	UUO	Primary podocytes	Downregulation of Wnt inhibitory factor-1	Promotes kidney fibrosis	[106]
FoxM1	UUO, FA	Ang II	Transcriptionally regulating multi-Wnts expressions	Promotes kidney fibrosis	[107]
Brahma-related gene-1	UUO	TGF-β, Etoposide	Inhibition of autophagy	Promotes tubular senescence and renal fibrosis	[108]
IKKα	UUO, IR	TGF-β	Enhanced β-catenin nuclear translocation	Aggravates renal fibrogenesis	[109]
Indoleamine-2,3-Dioxygenase	IR	No vitro models	Unknown	Inducing kidney fibrosis after AKI	[110]
miR-21	Aristolochic acid	Aristolochic acid	Unknown	Promotes AKI-CKD transition	[111]
lncRNA-H19	IR	TGF-β	Downregulation of miR-196a	Inducing kidney fibrosis	[112]

AKI, acute kidney injury; CKD, chronic kidney disease; TGF-β, transforming growth factor-beta; FoxM1, forkhead box protein M; IKK, inhibitor of kappa B kinase; IR, ischemia–reperfusion; UUO, unilateral ureteral obstruction, LRP5: low-density-lipoprotein receptor-related protein 5; Ang II, angiotensin II.

**Table 2:** Notch pathway in the repair of renal injury.

Receptors	Receptors’ Source	Ligands	Target Genes	Models	Mechanism	Influence	References
Notch 1	Vascular endothelial cells	Dll4	HEY1, HEY2, NOTCH3	HUVECs: combinant human DLL4 and recombinant human Jag1	EMT	Promoting renal fibrosis	[127]
Notch1	TECs	Dll1	Unknown	HK2/mice: cisplatin	Promoting kidney inflammation	Promoting renal injury	[128]
Notch1	TECs	Dll4	Unknown	Mice: IRI PTECs: hypoxia–reoxygenation	Causing prosenescence	Renal maladaptive repair	[129]
Notch1	TECs	Jagged-1	Hes-1 and Hey-1	Mice, TECs: Gremlin	Activation of NF-κB pathway	Proinflammation	[130]
Notch1	Macrophage	Dll4	Hes1, Hey1, and Hey2	RAW264.7: Indoxyl Sulfate	Interplay of OATP2B1	Proinflammation	[131]
Notch1	Macrophage	Unknown	IKK-B and p65	RAW 264.7 /mice: high glucose	M1 polarization	Promoting renal fibrosis	[122]
Notch2	TECs	Jagged-1	Hes-1	Mice: IRI	STAT3 phosphorylation and upregulation of survivin	Promoting functional and structural recovery	[126]
Notch2	TECs	Hes1	Unknown	Rats: IR	Promoting inflammation and apoptosis	Promoting renal injury	[132]
Notch3	TECs and podocyte	Unknown	Unknown	Mice: lipopolysaccharide	TLR4/NOTCH3	Renal apoptosis and inflammation	[133]

TECs: tubular epithelial cells; EMT, epithelial-to-mesenchymal transition; TLR4, Toll-like receptor 4; NF-κB, nuclear factor-kappa B; OATP2B1, organic anion transporting polypeptides2B1.

**Table 3:** The regulation of the Hedgehog pathway in kidney injury.

Regulator	In Vivo Models	In Vitro Models	Regulating Mechanisms	Influence on Renal	Reference
BPPs	UUO	TGF-β	Decreased E-cadherin expression	Ameliorating renal fibrosis and EMT	[140]
Kaempferol	Hypertensive	TGF-β	I nhibiting the activation of Shh and Gli through increasing the expression of Sufu	Inhibiting renal fibrosis and EMT	[138]
CDGSH iron sulfur domain 2	LPS	LPS	Unknown	Alleviating septic AKI	[135]
Hydroxytyrosol	IR	H/R	Inhibiting apoptosis	Inh i biting apoptosis	[136]

mTECs, mouse renal tubular epithelial cells; IR, ischemia–reperfusion; UUO, unilateral ureteral obstruction; PTECs, primary renal tubular epithelial cells; lps, lipopolysaccharide; Shh, Sonic Hedgehog; H/R, hypoxia–reoxygenation; BPPs, polysaccharides extracted from balanophora polyandra Griff; EMT, epithelial-to-mesenchymal transition.

**Table 4:** Emerging therapeutic strategies targeting AKI-CKD transition.

Mechanism	Examples	Advantages	Challenges	Current Stage
Exosomes	FRC exosomes	Suitable for delivery, with a wide range of sources, with a variety of contents Reducing inflammation and fibrosis Promoting tissue repair and regeneration	They lack tissue specificity and targeting, and the mixed effects of their contents are not clear, lacking long-term safety.	Preclinical research
Metabolic interventions	Small-molecule inhibitors of PFKFB3: 3PO	Improving renal energy metabolism Reducing inflammation and fibrosis	Drug side effects Individual differences Long-term efficacy and safety	More preclinical studies A few clinical trials (SGLT2)
Clearance of senescent cells	Combination of dasatinib and quercetin	Reducing inflammation and fibrosis Promoting tissue repair and regeneration Improving treatment outcomes and prognosis	Potential adverse reactions Clearance efficiency and specificity issues Adaptability to disease stages	More preclinical studies Less clinical data in age-related diseases

SGLT2, Sodium–Glucose Transporter 2; PFKFB3, 6-phosphofructo-2-kinase/fructose-2,6-biphosphatase 3; FRC, fibroblastic reticular cell.

## Data Availability

Not applicable.

## Abbreviations

AKI	Acute kidney injury
CKD	Chronic kidney disease
PTCs	Proximal tubular epithelial cells
GSDMD	Gasdermin D
IL-1β	Interleukin-1β
RIPK	Receptor-interacting protein kinase
MLKL	Mixed lineage kinase domain-like protein
TCF/LEF	T-cell factor/lymphoid enhancer factor
Notch1–4	Notch receptors
TCA	Tricarboxylic acid cycle
Dll	Delta-like
Jag	Jagged
Hh	Hedgehog
Shh	Sonic Hedgehog
Ihh	Indian Hedgehog
Dhh	Desert Hedgehog
Ptch/Smo	The primary transmembrane receptors comprise patched and smoothened
Havcr1/KIM1	Kidney Injury Molecule 1
Krt8	Keratin 8
Krt20	Keratin 20
Lcn2/NGAL	Neutrophil gelatinase-associated lipocalin
SOX9^+^	SOX9-positive
CDH6	Cadherin 6
NF-κB	Nuclear factor κB
CREs	Conserved cisregulatory elements
P21	P21CIP1
P16	P16INK4A/pRb
CDKS	Cyclin-dependent kinases
CKIs	Cyclin-dependent kinase inhibitors
ETC	Electron transport chain
DRP1	Dynamin-Related Protein 1
MFN1	Mitofusin 1
MFN2	Mitofusin 2
OPA1	Optic Atrophy 1
PGC-1α	Peroxisome proliferator-activated receptor gamma coactivator-1α
FAO	Fatty acid oxidation
ATP	Adenosine triphosphate
TFAM	Mitochondrial transcription factor A
PPARα	Proliferator-activated receptor α (PPARα)
KLF15	Krüppel-like factor 15
TGFβ	Transforming growth factor-beta
H2K4Me1	Histone 3 lysine 4 monomethylation
OXPHOS	Oxidative phosphorylation
